# Genomic, Functional, and Evolutionary Insights into a Novel T7-like Phage B1 Infecting Multidrug-Resistant *Enterobacter cloacae*

**DOI:** 10.3390/ijms27010195

**Published:** 2025-12-24

**Authors:** Yun-Chan Tsai, Soon-Hian Teh, Philip Huang, Ling-Chun Lin, Nien-Tsung Lin

**Affiliations:** 1 Master Program in Biomedical Sciences, School of Medicine, Tzu Chi University, No. 701, Sec. 3, Zhongyang Road, Hualien 97004, Taiwan; jim.tsai201248@gmail.com; 2 Division of Infectious Diseases, Department of Internal Medicine, Hualien Tzu Chi Hospital, Buddhist Tzu Chi Medical Foundation, No. 707, Sec. 3, Zhongyang Road, Hualien 97004, Taiwan; jimmyteh2000@gmail.com; 3 Department of Microbiology and Immunology, School of Medicine, Tzu Chi University, No. 701, Sec. 3, Zhongyang Road, Hualien 97004, Taiwan; 111311112@gms.tcu.edu.tw

**Keywords:** antimicrobial resistance, bacteriophage, *Enterobacter cloacae*, multidrug resistance, phage therapy, T7-like phage

## Abstract

Multidrug-resistant (MDR) *Enterobacter cloacae* is a growing public health issue worldwide, highlighting the urgent need for alternative antimicrobial strategies. This study reports on a lytic phage, designated B1, isolated from sewage, which exhibits specificity and lytic efficiency against MDR *E. cloacae*. Morphological observation revealed that B1 possesses an icosahedral head (~54 nm) and a short tail (~13 nm). Phage B1 showed a narrow host range, demonstrated stability within a temperature range of 4–37 °C, tolerance to pH values between 5 and 11, and showed an excellent bacteriolytic capacity with a short latent period of less than 10 min and a burst size of approximately 150 PFU/initially infected cell, indicating a rapid lytic cycle and efficient replication capability. Whole-genome sequencing revealed that the phage genome consists of 40,163 base pairs of double-stranded DNA containing 52 open reading frames (ORFs) with a GC content of 52%. Comparative genome-wide analysis using VIRIDIC revealed that B1 shares 75% to 92% similarity with *Escherichia* phage IMM-002 (accession: NC_048071), *Citrobacter* phage SH4, and *Cronobacter* phage Dev2 (accession: NC_023558), but shares less than 70% similarity with other *Enterobacter* phages. According to ICTV criteria, B1 represents a new species within the same genus as T7-like phages belonging to *Autographiviridae*, subfamily *Studiervirinae*, genus *Kayfunavirus*. In addition, B1 lacks lysogeny-associated or virulence genes and exhibits potent lytic activity against multidrug-resistant *E. cloacae*, making it a promising candidate for phage therapy. These findings opened up our understanding of the diversity of T7-like phages and provided insights into their evolutionary adaptability and therapeutic potential.

## 1. Introduction

Multidrug-resistant (MDR) bacteria pose a significant threat to public health and clinical medicine. The World Health Organization (WHO) has listed carbapenem-resistant *Enterobacteriaceae* as a priority for developing new antimicrobial strategies [1]. *Enterobacter cloacae*, a member of the *Enterobacter cloacae* complex (ECC), is a facultatively anaerobic, Gram-negative bacterium that forms part of the normal gut microbiota in humans but can act as an important opportunistic pathogen that can cause various infections, including bloodstream infections, respiratory infections, urinary tract infections, wound infections, and septicemia, particularly in immunocompromised individuals [2]. The global emergence of *E. cloacae* that is resistant to β-lactams, carbapenems, and aminoglycosides has significantly reduced the effectiveness of conventional antibiotic therapies, leading to prolonged hospitalization, increased costs, and higher mortality rates [3]. According to data from the Taiwan Antimicrobial Resistance Surveillance (TSAR) Program, the nonsusceptibility rate of *Enterobacteriaceae* to third- and fourth-generation cephalosporins (cefotaxime and cefepime) remained relatively stable. However, the nonsusceptibility rate to piperacillin/tazobactam increased significantly, from 8.8% in 2006 to 21.4% in 2020. In contrast, the nonsusceptibility rate to imipenem was initially high between 2002 and 2006 (12.5–15.4%), decreased significantly between 2010 and 2014 (0–3.3%), and then increased again to 14.3% in 2020 [4]. Therefore, alternative therapeutic approaches other than conventional antibiotics underscore the urgent need.

Bacteriophages (phages), viruses that specifically infect bacteria, have attracted renewed attention as potential therapeutic agents against MDR bacteria. Phages offer several advantages over traditional antibiotics, including high host specificity, self-amplification at the site of infection, and minimal disruption to the host microbiota [5]. In recent years, both preclinical and clinical studies have demonstrated the feasibility of phage therapy in treating infections caused by MDR Gram-negative bacteria such as *Pseudomonas aeruginosa*, *Acinetobacter baumannii*, *Klebsiella pneumoniae*, and *E. coli* [6,7,8,9]. Among the diverse phage families, members of the *Autographiviridae* family, particularly T7-like phages, are of particular interest due to their well-characterized lytic life cycle, rapid replication, and relatively simple genome organization, which facilitate genetic and functional studies [10].

Despite the clinical importance of *E. cloacae*, relatively few lytic phages targeting this pathogen have been characterized in detail. Most reported *E. cloacae* phages belong to the myovirus or siphovirus [11,12,13,14,15], while T7-like podophages against this species remain largely unexplored. In this study, we isolated and characterized a novel lytic T7-like phage, designated vB_EclP_B1 (abbreviated as B1), from a wastewater sample collected in Taiwan. Phage B1 demonstrated strong lytic activity against an MDR *E. cloacae* clinical isolate and exhibited a typical podovirus morphology under transmission electron microscopy. We performed whole-genome sequencing and comprehensive bioinformatics analyses to elucidate its genomic features and assess its therapeutic potential. Our findings expand the current understanding of T7-like phages infecting *E. cloacae* and highlight the potential of B1 as a candidate for phage therapy targeting MDR *E. cloacae* infections. This is among the few studies to report a T7-like phage specifically targeting MDR *E. cloacae*, making it a valuable addition to the current phage research landscape.

## 2. Results

### 2.1. Isolation, Purification, Morphology, and Host Range Analysis of vB_EclP_B1 (B1)

*E. cloacae* clinical isolate ECL51159 exhibits multidrug resistance to β-lactam antibiotics, including amoxicillin/clavulanic acid, ampicillin, cefmetazole, and cefazolin. The increasing prevalence of β-lactam-resistant *E. cloacae* strains [16] highlights the urgent need for alternative treatment options, underscoring the importance of isolating and characterizing novel lytic phages for their potential clinical applications.

**Figure 1:**
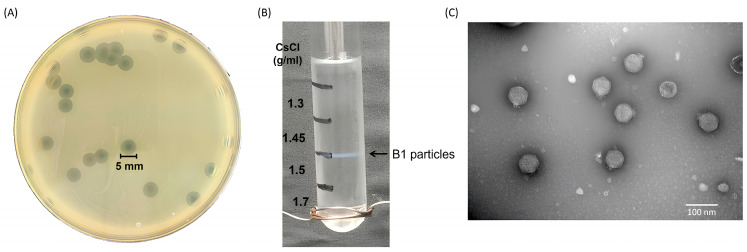
Morphological characterization and purification of phage B1. (**A**) Plaque morphology of phage B1 on *E. cloacae* lawn after 18 h incubation at 37 °C, showing clear plaques with surrounding halo zone. (**B**) Purification of B1 particles by CsCl density gradient ultracentrifugation, showing a distinct blue band corresponding to the phage layer. (**C**) Transmission electron micrograph of purified B1 virions, revealing an icosahedral head and a short non-contractile tail, consistent with the morphology of members of podovirus (scale bar = 100 nm).

Phage vB_EclP_B1 (hereafter referred to as B1) was isolated from hospital wastewater collected near Buddhist Tzu Chi Hospital, using *E. cloacae* strain ECL51159 as the host. When plated on a bacterial lawn, B1 formed small, clear plaques (<1 mm in diameter) surrounded by prominent halos of approximately 5 mm (Figure 1A). These halos continued to expand at a rate of 2–3 mm per day, indicating that the phage likely possesses capsular polysaccharide-degrading activity.

For further characterization, B1 was enriched to prepare a high-titer stock of 10^11^ PFU/mL, which was then subjected to ultracentrifugation in a discontinuous cesium chloride (CsCl) gradient (ρ = 1.7, 1.5, 1.45, and 1.3 g/mL). Phage particles banded at the interface between 1.5 and 1.45 g/mL (Figure 1B). Transmission electron microscopy (TEM) revealed that B1 has an icosahedral head of approximately 54 nm in diameter and a short tail of approximately 13 nm in length (Figure 1C), consistent with morphological features of podophages.

The host range of B1 was evaluated by spot testing against 128 clinical isolates of *E. cloacae* as well as clinical isolates of *K. pneumoniae*, *P. aeruginosa*, *E. coli*, and *A. baumannii*. None of the tested strains were susceptible to infection by B1, indicating that this phage exhibits an extremely narrow host range.

### 2.2. Replication Kinetics and Lytic Activity of B1

**Figure 2:**
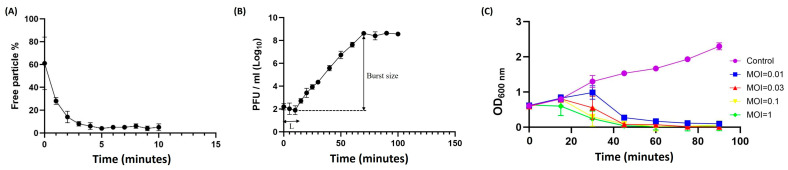
Adsorption kinetics, infection dynamics, and bactericidal activity of phage B1 against *E. cloacae* ECL59115. (**A**) Adsorption assay showing that approximately 87% of phage B1 particles were adsorbed onto host cells within 2 min, increasing to over 96% by 5 min, indicating rapid attachment efficiency. (**B**) Infection dynamics of phage B1 monitored in a one-step growth-type assay performed at an MOI of 0.001. A decrease in PFU was observed during the early phase, followed by an increase after approximately 10 min, representing the first detectable burst. The estimated burst size, calculated from this initial rise in PFU, was approximately 150 PFU/initially infected cell after 3–4 burst steps. (**C**) Bacterial growth inhibition curves of phage B1 at different MOIs (0.01, 0.03, 0.1, and 1). While the optical density (OD_600_) of the untreated control increased steadily, phage-treated cultures showed a marked decline in turbidity beginning around 40 min, demonstrating strong bacteriolytic activity across multiple MOIs.

Phage B1 exhibited rapid adsorption to *E. cloacae* ECL59115, with approximately 87% of particles attached within 2 min and more than 96% adsorbed after 5 min (Figure 2A). A one-step growth-type assay conducted at an MOI of 0.001 revealed an initial decline in free phage titer followed by an increase after roughly 10 min, indicating the timing of the first detectable burst event (Figure 2B). Based on this initial rise in PFU, the burst size was approximately 150 PFU/initially infected cell after 3–4 burst steps; however, this value should be interpreted with caution, as secondary infection cycles may occur at MOIs greater than 0.001. Despite this limitation, the infection pattern aligns with the replication dynamics reported for other T7-like *Autographiviridae* phages. In addition, B1 exhibited strong bacteriolytic activity across multiple MOIs, with a marked reduction in culture tur131bidity beginning around 40 min post-infection (Figure 2C), demonstrating its rapid and potent killing activity against *E. cloacae*.

### 2.3. Environmental Stability of the B1

**Figure 3:**
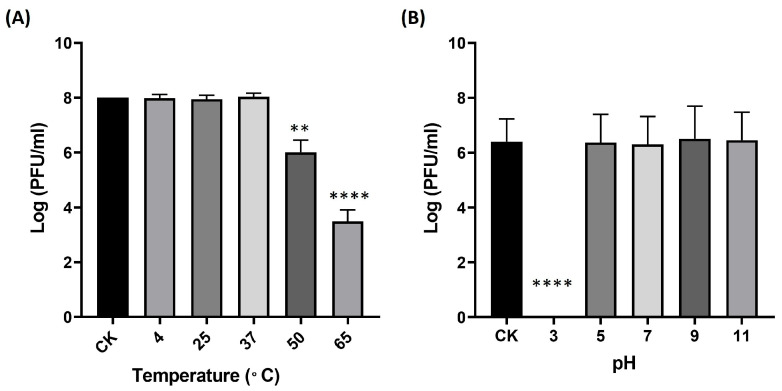
Environmental stability of B1 under different temperature (**A**) and pH (**B**) conditions. Data points represent the Mean  ±  SD of three biological replicates. The originally loaded titer is indicated as CK. Asterisks indicate significant differences compared to CK (** *p* ≤ 0.01; **** *p* ≤ 0.0001).

Environmental parameters—particularly temperature and pH—are known to strongly influence the structural stability and infectivity of phages [17]. For clinical or biotechnological applications, phage viability may be compromised by fluctuations during production, storage, transportation, and delivery. Therefore, assessing the thermal and pH stability of phage B1 is essential for defining appropriate storage conditions and formulation strategies. Following a 1 h incubation at 4 °C, 25 °C, and 37 °C, the titer of phage B1 showed no significant change relative to the initial concentration. In contrast, exposure to higher temperatures resulted in pronounced losses of infectivity, with a reduction of approximately 2 log units at 50 °C and 5 log units at 65 °C, indicating that B1 is heat-sensitive and can be classified as thermolabile (Figure 3A). To further evaluate long-term storage stability, crude B1 lysates (~10^8^ PFU/mL) were maintained at 4 °C in SM buffer and assayed monthly. Over a 12-month period, no meaningful decline in infectious titer was detected, demonstrating that B1 remains highly stable under refrigerated storage conditions.

To evaluate the pH stability of phage B1, the phage was incubated in LB medium adjusted to various pH values at 37 °C for 1 h, after which its infectivity was determined by plaque assay. The results showed that B1 retained full infectivity across a broad pH range from 5 to 11, which encompasses the physiologically relevant pH conditions encountered in most human tissues (approximately pH 6.5–7.4). In contrast, a drastic reduction in phage titer was observed at pH 3 (*p* < 0.0001), indicating that B1 is highly sensitive to strongly acidic environments (Figure 3B).

### 2.4. Genome Size Determination and Restriction Analysis of Phage DNA

**Figure 4:**
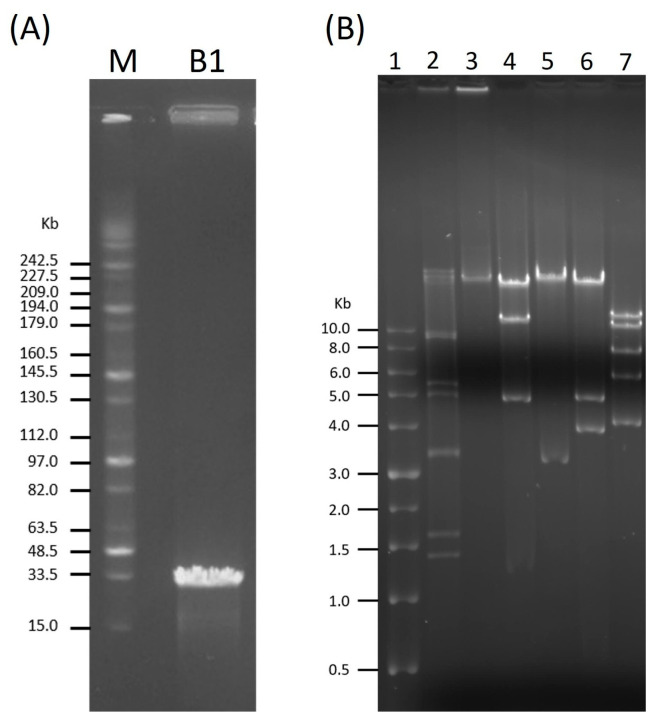
Genome size estimation and restriction analysis of phage B1. (**A**) Pulsed-field gel electrophoresis (PFGE) analysis showing the estimated genome size of phage B1 (~34 kb). Lane M: MidRange PFG Marker (New England BioLabs, Ipswich, MA, USA). (**B**) Restriction digestion profiles of B1 genomic DNA. Lane 1: TriDye^TM^ 1 kb DNA Ladder (New England BioLabs); lane 2: λ DNA–HindIII Digest (New England BioLabs); lane 3: undigested B1 DNA as a control; lanes 4–7: B1 genomic DNA digested with EcoRV, EcoRI, HindIII, and MluI, respectively.

The genome size of B1 was found to be approximately 34 kb via PFGE (Figure 4A). Subsequently, restriction digestion using EcoRV, EcoRI, HindIII, and MluI produced multiple distinct DNA fragments (Figure 4B), confirming that B1 possesses a double-stranded DNA genome susceptible to restriction endonuclease cleavage.

### 2.5. Genome Annotation and Functional Analysis

**Figure 5:**
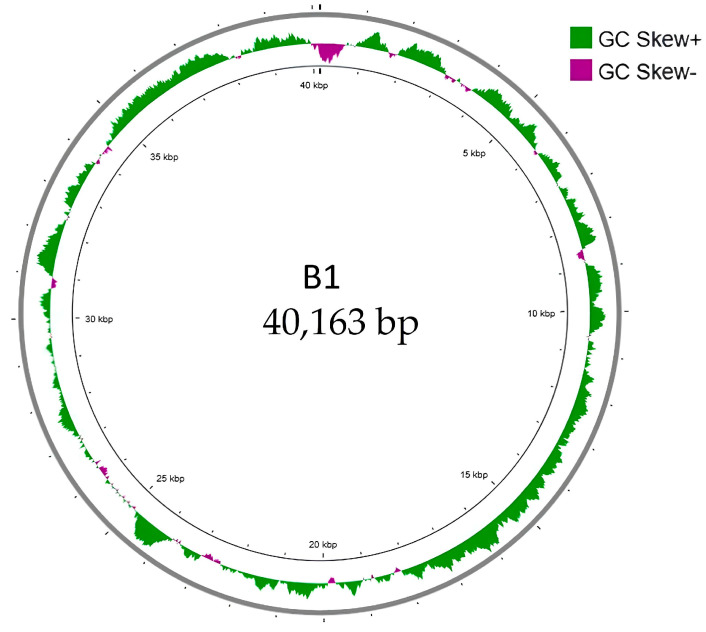
GC skew analysis of phage B1 genome. The circular map illustrates the GC skew pattern of phage B1 across its 40,163 bp linear double-stranded DNA genome. GC skew was calculated as (G − C)/(G + C) and plotted cumulatively, with positive skew values shown in green and negative skew values shown in purple. The distribution of GC skew reveals regions of strand bias that correspond to replication- and transcription-related asymmetry within the genome. No pronounced shift in GC skew polarity was observed, consistent with a compact, unidirectional genome organization typical of T7-like phages.

Whole-genome sequencing revealed that B1 contains a linear double-stranded DNA with a genome size of 40,163 base pairs with a GC content of 52%, comparable to that of *E. cloacae* ATCC 13047 (54.79%) [18]. After conducting a BlastN analysis, we found that the B1 genome was closely related to *Enterobacter* phage EP1 (accession: OM457002) [19] with 87% query coverage and 91.63% similarity and to *Citrobacter* phage SH4 (accession: NC_031018) [20] with 81% query coverage and 91.6% similarity. Overall, the genome architecture and similarity to other phage genomes suggested that phage B1 was similar to T7-like phages. GC skew analysis was performed to examine the nucleotide composition asymmetry and potential replication orientation of the phage B1 genome (Figure 5). The plot revealed alternating regions of positive and negative GC skew, represented by green and purple peaks, respectively. This pattern suggests strand-specific nucleotide bias commonly associated with transcriptional polarity rather than a distinct bidirectional replication origin, consistent with the linear and highly compact genomic organization typical of T7-like phages, which replicate via a unidirectional mechanism initiated near the terminal repeat region [21].

**Figure 6:**
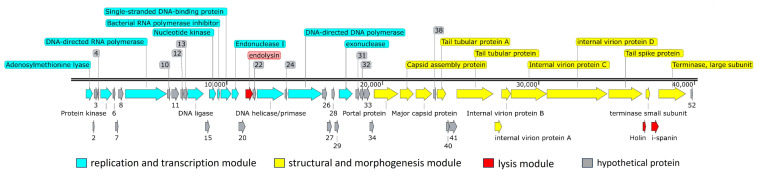
Genome organization of phage B1. The genome of phage B1 is arranged into three major functional modules: the replication and transcription module (blue), structural and morphogenesis module (yellow), and lysis module (red). Hypothetical proteins with no assigned function are shown in gray. Arrows indicate predicted open reading frames (ORFs), with the numbers corresponding to ORF numbers as listed in Table 1, and the arrow direction representing the direction of transcription. The scale bar denotes genome length in base pairs. The modular layout is characteristic of *Autographiviridae* phages, reflecting a streamlined genome organization optimized for efficient host takeover, virion assembly, and cell lysis.

A total of 52 open reading frames (ORFs) were predicted in the genome of phage B1 using RAST, all of which were located on the same DNA strand. Among these, 26 ORFs were assigned putative functions based on BlastP homology, whereas the remaining were annotated as hypothetical proteins with no significant matches in current databases (Table 1). The predominant start codon was ATG (90.4%), followed by GTG (3.8%), TTG (3.8%), and GAA (1.9%). Stop codons included TAA (73%), TGA (25%), and TAG (2%). The annotated ORFs were organized into functional modules, consistent with a typical T7-like genome architecture (Figure 6). Notably, ORF1 encodes an adenosylmethionine lyase, an enzyme involved in the S-adenosylmethionine (SAM) cycle that cleaves S-adenosylhomocysteine (SAH) into adenosine and homocysteine [22]. The presence of this gene suggests that B1 may modulate host methylation dynamics by preventing SAH accumulation, thereby maintaining methyltransferase activity and supporting phage DNA modification processes during infection.

The replication and transcription module includes genes encoding proteins involved in nucleotide metabolism, DNA replication, and transcriptional regulation, such as ORF14 (DNA ligase), ORF16 (nucleotide kinase), ORF18 (single-stranded DNA-binding protein), ORF23 (DNA helicase/primase), ORF25 (DNA polymerase), and ORF30 (exonuclease). A phage-encoded RNA polymerase (ORF9) was identified, a hallmark of the *Autographiviridae* family, enabling autonomous transcription of phage genes independent of host RNA polymerase. Additionally, ORF17 encodes a bacterial RNA polymerase inhibitor, suggesting an early host takeover mechanism that redirects transcription toward phage replication.

The structural and morphogenesis module of phage B1 comprises genes involved in virion assembly and host recognition, including ORF35 (portal protein), ORF36 (capsid assembly protein), ORF37 (major capsid protein), ORFs 39 and 42 (tail tubular proteins), ORFs 43–46 (internal virion and tail-associated proteins), ORF47 (tail spike protein), and ORFs 49 and 51 (small and large terminase subunits responsible for DNA packaging). ORFs 43–46 contain conserved domains characteristic of *Autographiviridae* (PHA00432, PHA00101, PHA00431, and PHA00368), reflecting their roles in forming the short, non-contractile tail, stabilizing the tail structure, and initiating host adsorption.

Further analysis of ORF47 revealed that it encodes a multifunctional tail fiber/spike protein with three conserved regions: an N-terminal tail fiber structural domain (PHA00430, residues 1–112) responsible for anchoring to the baseplate, a Tail_spike_N domain (pfam18668, residues 147–207) associated with receptor recognition, and a Pectate_lyase_3 domain (pfam12708, residues 231–349) linked to capsular polysaccharide degradation. This domain architecture indicates that B1 is capable of both specifically binding to its *Enterobacter* host and enzymatically degrading the capsular barrier, thereby enhancing adsorption efficiency and promoting successful genome injection. Such capsule-targeting features are considered key determinants of host range and infection efficiency among T7-like *Autographiviridae* phages [23].

A lysis cassette was identified in the B1 genome, composed of endolysin (ORF21), holin (ORF48), and i-spanin (ORF50), which function cooperatively to mediate host cell lysis and phage progeny release. ORF21 encodes a peptidoglycan lytic transglycosylase, which hydrolyzes the bacterial cell wall during the final stage of infection. ORF48, annotated as a type II holin (PHA00426), contains a conserved *Phage*_*holin*_*2*_*2* domain and is a hallmark of small, hydrophobic membrane proteins that accumulate in the cytoplasmic membrane and act as molecular timers to trigger lysis [24]. ORF50 encodes an inner membrane spanin (i-spanin), which likely cooperates with holin and endolysin to complete the lytic process by disrupting the outer membrane [25]. Together, these three genes form a functionally complete lysis module supporting the rapid and efficient lytic phenotype observed for phage B1.

No tRNA genes were detected in the genome, indicating that phage B1 relies entirely on the host’s translational machinery. Moreover, no antibiotic resistance genes or virulence factors were identified using ResFinder and VirulenceFinder analyses, underscoring its biosafety and potential suitability for therapeutic application.

**Figure 7:**
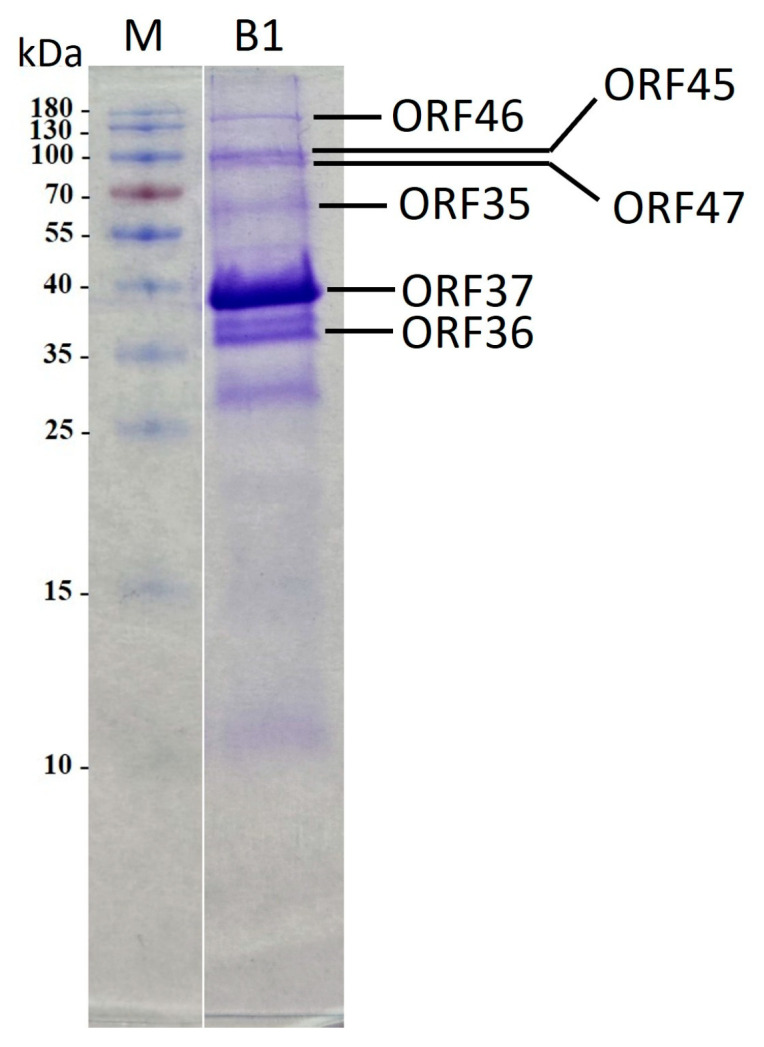
Sodium dodecyl sulphate-polyacrylamide gel electrophoresis of B1 virion proteins. Lanes: M, PageRulerTM Prestained Protein Ladder (Thermo Fisher Scientific); B1, phage B1 structural proteins. Relative migrations of molecular mass marker proteins are indicated on the left. ORF identified via MS/MS are indicated on the right.

Furthermore, a virion protein analysis was conducted via SDS-PAGE. The findings revealed three prominent protein bands alongside approximately ten minor protein bands, exhibiting diverse molecular weights spanning from 10 to 180 kDa (Figure 7). Subsequently, eight protein bands were isolated and subjected to MS/MS analysis. The outcomes corroborated the genomic annotations of six structural proteins, including ORFs 35 to 37 and ORFs 45 to 47, as the predictors of portal protein, capsid assembly protein, major capsid protein, internal virion protein C, internal virion protein D, and tail spike protein. Notably, certain putative structural proteins remained unidentified through this method, likely attributable to their relatively low abundance.

### 2.6. Comparative Genomics and Phylogenetic Analysis

**Figure 8:**
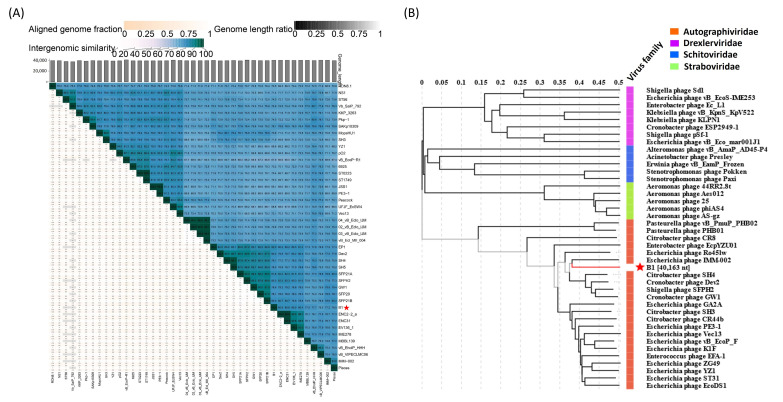
Comparative genomic relationships and taxonomic placement of phage B1. (**A**) Intergenomic similarity heatmap generated by VIRIDIC, showing percentage nucleotide identity, aligned genome fraction, and genome length ratio among B1 and representative *Autographiviridae* phages. Asterisks indicate phage B1, which clusters with *Escherichia* phage IMM-002, *Citrobacter* phage SH4, and *Cronobacter* phage Dev2, indicating strong genomic relatedness. (**B**) Genome-based phylogenetic tree displaying the taxonomic placement of B1 among *Autographiviridae* (orange), *Drexlerviridae* (purple), *Schitoviridae* (blue), and *Straboviridae* (green). Phage B1 (marked by an asterisk) forms a monophyletic group with IMM-002, SH4, and Dev2, consistent with its categorization as a T7-like lytic phage.

To determine the taxonomic relationship of phage B1 with previously characterized phages, pairwise intergenomic similarities were calculated using VIRIDIC [26]. The resulting heatmap revealed that B1 forms a cluster with *Escherichia* phage IMM-002 (accession: NC_048071), *Citrobacter* phage SH4, and *Cronobacter* phage Dev2 (accession: NC_023558), sharing ~75–92% nucleotide identity across large portions of the genome. This high degree of similarity, together with >0.8 aligned genome fraction and similar genome length ratios, supports that these phages belong to a closely related lineage within *Autographiviridae* (Figure 8A). In addition, the genome-based phylogenetic tree of B1 comparison by VIPtree showed that B1 groups tightly with IMM-002, SH4, and Dev2, forming a well-supported monophyletic branch distinct from phages infecting *Pseudomonas*, *Acinetobacter*, and *Aeromonas* (Figure 8B).

**Figure 9:**
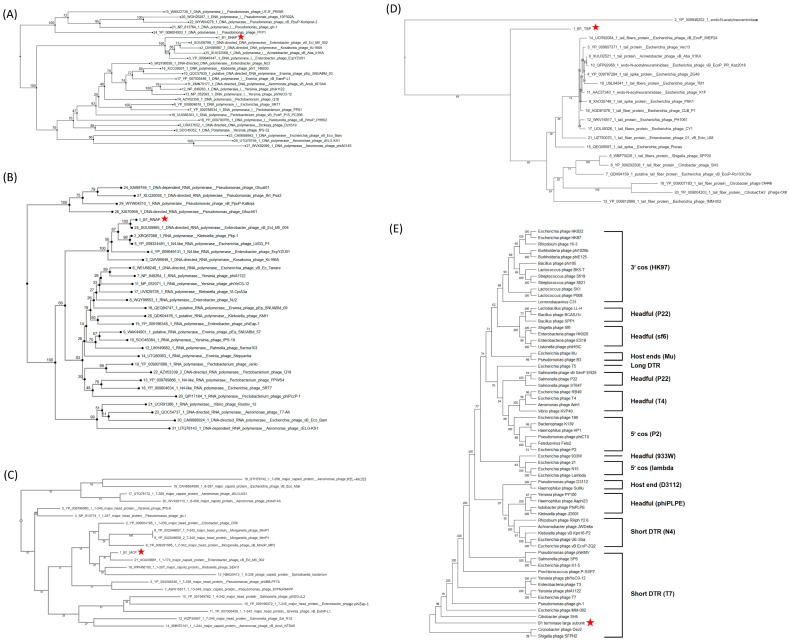
Phylogenetic analyses of key functional proteins of phage B1. Neighbor-joining phylogenetic trees were constructed for (**A**) DNA-directed DNA polymerase, (**B**) DNA-directed RNA polymerase, (**C**) major capsid protein, (**D**) tail spike protein, and (**E**) terminase large subunit, based on amino acid sequences. Red stars indicate the position of phage B1 in each tree. In panels (**A**–**D**), B1 clusters closely with *Enterobacter* and *Citrobacter* phages belonging to the *Autographiviridae* family, supporting its classification within this lineage. In panel (**E**), based on terminase large subunit phylogeny, B1 clusters within the short direct terminal repeat (DTR) clade characteristic of T7-like phages, consistent with its genome organization and predicted DNA packaging strategy. Numbers at the nodes indicate bootstrap support values. Brackets denote different DNA packaging strategies, with representative phages for each strategy indicated in parentheses. Bootstrap support values (>50%) are shown at key branching nodes.

A DNA polymerase I-based phylogenetic tree grouped B1 with *Enterobacter* phage vB_Ecl_MII_002 (accession: XGU09798), and EcpYZU01 (accession: YP_009949147), *Acinetobacter* phage vB_Aba_01KA (accession: XUU32568) and *Kosakonia* phage Kc166A (accession: QVV96867), confirming its evolutionary affinity with T7-like phages (Figure 9A). Similarly, an RNA polymerase-based phylogenetic tree revealed a highly supported monophyletic clade (bootstrap = 100) consisting of B1, *Enterobacter* phage vB_Ecl_MII_004 (accession: XGU09965), and *Klebsiella* phage Pkp-1 (accession: XRQ67088), indicating that B1 retains a consensus transcriptional module typical of T7-like phages (Figure 9B). A phylogenetic tree of major capsid proteins (MCPs) placed B1 alongside *Enterobacter* phage vB_Ecl_MII_002 (accession: XGU09881) and *Klebsiella* phage SEA1f (accession: WPH66100), supporting its conserved structural modules consistent with members of the *Autographiviridae* family (Figure 9C). In contrast, phylogenetic analysis of the tail spike protein (TSP) revealed that B1 formed a separate branch, distinct from *Shigella* phage SFP20 (accession: WBF70026) and related *Enterobacteriaceae* phages, highlighting a distinct receptor binding module that may underlie its specific infectivity against *E. cloacae* (Figure 9D). Phylogeny of the large subunit of terminase (TerL) provides additional insights into B1’s genome packaging strategy. B1 clusters with *Cronobacter* phage Dev2 (accession: YP_009005154) and *Shigella* phage SFPH2 (accession: YP_009807457) within the “short direct terminal repeat (DTR)” clade, suggesting that it employs a T7-type packaging mechanism characterized by precise cleavage and packaging of DNA containing short terminal repeats (Figure 9E). This finding further supports B1’s placement within the *Autographiviridae* family and is consistent with its overall genome organization.

**Figure 10:**
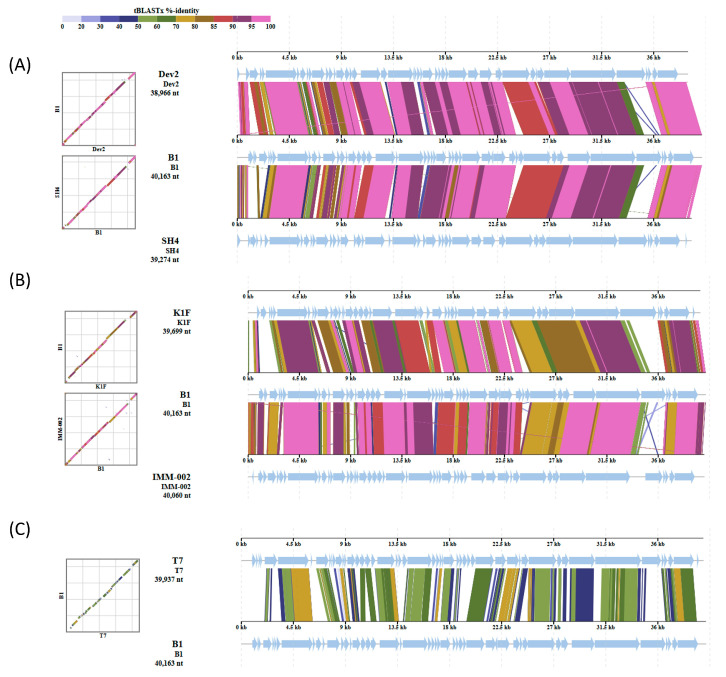
Comparative genomic analysis of phage B1 with related T7-like phages. Whole-genome tBLASTx version 2.17.0alignments show that B1 shares a conserved genomic backbone with phages Dev2 and SH4 (**A**); K1F and IMM-002 (**B**); and T7 (**C**). Arrows indicate predicted open reading frames (ORFs), and the arrow direction representing the direction of transcription. Core replication and structural gene regions display high synteny, while divergence is mainly observed in the tail spike pritein, consistent with variation in host recognition functions among *Autographiviridae* phages.

Furthermore, comparative genome alignments of phage B1 with closely related T7-like phages, including *Cronobacter* phage Dev2, *Citrobacter* phage SH4, *Escherichia* phage K1F (accession: NC_007456) and IMM-002, and the canonical *Escherichia* phage T7, revealed a highly conserved genomic organization (Figure 10). B1 shared extensive collinearity and high amino acid identity (>85% across most coding regions) with Dev2, SH4, K1F and IMM-002, particularly within the replication, transcription, and morphogenesis modules. In contrast, reduced similarity was observed in the tail spike gene region (corresponding to ORF47 in B1), where blocks of lower identity and altered synteny were detected, indicating divergence within the host recognition and adsorption module. Alignment with the model phage T7 showed overall conserved genomic structure but lower regional sequence identity, reflecting evolutionary distance within the *Autographiviridae* family.

## 3. Discussion

*E. cloacae* has emerged as an important nosocomial pathogen, frequently associated with bloodstream and urinary tract infections, particularly among immunocompromised individuals and those in intensive care units [1]. The increasing prevalence of carbapenem-resistant and extended-spectrum β-lactamase (ESBL)-producing *E. cloacae* strains has become a major global health concern [16]. Conventional antibiotic therapy is often ineffective, as resistance, commonly to β-lactam antibiotics, including ampicillin and amoxicillin/clavulanic acid, can lead to severe outcomes such as bacteremia, endocarditis, and increased mortality risk [27]. In Taiwan, national surveillance data from the SMART program (2009–2019) reported ceftriaxone resistance in nearly one-third of *Enterobacterales* isolates, with *E. cloacae* ranking among the top three resistant species [28]. Such alarming trends highlight the urgent need for alternative therapeutic approaches, and phage-based therapy is increasingly recognized as a viable option for targeting multidrug-resistant (MDR) *Enterobacter* infections [29].

In this study, a novel lytic phage, designated B1, was isolated from hospital wastewater in Hualien, Taiwan, and characterized as a T7-like phage within the *Autographiviridae* family. B1 displayed distinct biological and genomic features that differentiate it from previously described *E. cloacae* phages [13,14,15,30,31]. Phenotypically, B1 demonstrated rapid adsorption kinetics, achieving approximately 96% adsorption within 5 min, together with a short latent period and an estimated burst size of 153 PFU/infected cell. These traits indicate highly efficient replication dynamics consistent with virulent *Autographiviridae* phages. B1 also demonstrated broad pH stability (pH 5–11) and retained infectivity at 4 °C, suggesting a physicochemically robust nature favorable for storage, formulation, and therapeutic application. Collectively, these biological characteristics highlight the potential of B1 as a promising candidate for therapeutic development against MDR *E. cloacae*.

The burst size of B1 (~150 PFU/initially infected cell) falls within the characteristic range reported for well-studied T7-like phages. For example, the T7-like phage ϕIBB-PF7A, which infects *P. fluorescens*, exhibits a burst size of approximately 153 PFU/infected cell with a latent period of 15 min [32], while the classical coliphage T7 yields 179 ± 19 PFU/infected cell under optimal laboratory conditions [33]. These findings support the broader observation that highly productive T7-like phages often generate burst sizes between 120 and 180 PFU/infected cell. Nevertheless, notable variation has been documented among other T7-like podoviruses, with some phages exhibiting substantially lower burst sizes depending on host species, bacterial physiological state, and environmental conditions; for example, a burst size of only 44 PFU per infected cell has been reported for a T7-like phage infecting *P. aeruginosa* [34]. Such variability highlights that burst size is not a fixed trait within this group but rather a parameter shaped by both phage-specific factors and infection context. Within this comparative framework, the replication performance of B1 can be considered robust and broadly comparable to that of high-performing T7-like phages. The combination of rapid adsorption kinetics, a short latent period, and strong bacteriolytic activity further underscores its potential utility in therapeutic or anti-Enterobacter applications, particularly in settings where rapid bacterial reduction is required.

While this study provides a comprehensive assessment of the biological characteristics of phage B1, several methodological considerations should be acknowledged. The one-step growth assay was conducted at a low MOI (0.001), which may allow secondary rounds of infection and extend the apparent timing of lysis, particularly in the presence of heterogeneity in susceptibility across clinical *Enterobacter* isolates. Classical Ellis and Delbrück assay [35] conditions typically involve high MOI to achieve synchronous entry; however, repetition of such experiments was restricted by biosafety regulations associated with the clinical host strain. As a result, the lysis values reported here should be interpreted as approximations derived from the first detectable lysis cycle rather than a strictly synchronized, single-cycle event. Despite this limitation, the measured latency and lysis parameters align well with reported values for related *Autographiviridae* phages [32,33,34], supporting the reliability of the biological interpretations drawn from these experiments. These considerations do not alter the overall conclusions regarding the lytic characteristics of B1 but are noted to ensure methodological transparency.

Genomic analysis revealed that B1 possesses a linear double-stranded DNA genome of 40,163 bp with a GC content of 52%, comparable to that of its host *E. cloacae* (54.79%). The overall genome architecture conforms to the modular organization typical of T7-like phages, encompassing distinct functional regions for replication, morphogenesis, and lysis. However, B1 exhibits several unusual genomic features that distinguish it from canonical T7-like phages and suggest evolutionary diversification.

The most striking difference is the presence of ORF1, encoding an adenosylmethionine lyase, a gene previously identified in bacteriophage T3 as a counter-defense mechanism against bacterial restriction–modification systems [22], but rarely reported in the *Autographiviridae* family. This enzyme functions in the SAM cycle, catalyzing the conversion of SAH into adenosine and homocysteine, thereby preventing the accumulation of SAH that inhibits methyltransferase activity [36]. The occurrence of this gene in phage B1 implies that it may actively modulate the SAM pathway to sustain an optimal methylation potential, promoting phage DNA modification and protection from host restriction enzymes. Because SAM-dependent methylation plays a central role in bacterial epigenetic regulation and restriction defense, the acquisition of an adenosylmethionine lyase by B1 likely represents a metabolic adaptation enhancing its replication in hosts with active restriction barriers. Such integration of host-like metabolic functions is uncommon among T7-like phages and may have arisen through horizontal gene transfer from bacterial hosts, conferring a selective advantage during infection.

Another defining feature of B1 lies in ORF47, which encodes a multifunctional tail fiber/spike protein containing both receptor-binding and enzymatic domains. Domain analysis identified an N-terminal structural domain (PHA00430) for anchoring to the baseplate, a Tail_spike_N region (pfam18668) responsible for receptor recognition, and a Pectate_lyase_3 domain (pfam12708) associated with capsular polysaccharide degradation [37]. This domain organization implies that B1 possesses dual capabilities: specific recognition of host surface receptors and enzymatic degradation of the bacterial capsule. Such capsule-degrading activity is rarely documented among *E. cloacae* phages. The coexistence of receptor-binding and pectate lyase domains suggests that B1 evolved a specialized infection strategy targeting encapsulated *E. cloacae*, allowing it to breach the host’s protective barrier for efficient genome delivery. This structural innovation likely contributes to its strong lytic performance and narrow host range, representing a hallmark of host-adapted specialization within the T7-like lineage. The presence of similar enzymatically active tail spikes has been reported in *Acinetobacter* phages that degrade capsular polysaccharides [23,38,39], supporting the idea that capsule-targeting phages evolve convergently to overcome polysaccharide-mediated resistance mechanisms.

A notable feature is that in many phages, especially double-stranded DNA tailed phages (*Caudoviricetes*), the genes responsible for host cell lysis are typically organized in clustered gene modules. However, this arrangement is not universal [40]. In some phages, such as some T7-like and phiKMV-like phages, the lysis modules can be split or rearranged, as seen in phage B1. In this phage, the lysis-associated genes—endolysin (ORF21), holin (ORF48), and i-spanin (ORF50)—are distributed across the genome rather than forming a single operon. This dispersed gene organization implies a distinct regulatory mechanism governing lysis timing. In canonical phages, lysis genes are typically co-transcribed to coordinate host cell wall degradation and the release of progeny virions [25]. In contrast, the separated lysis genes in B1 may enable more flexible or independently controlled expression, allowing for precise temporal regulation under different infection conditions. Such a non-contiguous arrangement likely reflects evolutionary genome rearrangements that enhance infection efficiency or adaptability to specific bacterial hosts.

Comparative genomic analysis using VIRIDIC [26] revealed that B1 shares 85–92% intergenomic similarity with Enterobacter phage IMM-002, Citrobacter phage SH4, and Cronobacter phage Dev2—values below the 95% species demarcation threshold defined by ICTV—indicating that B1 represents a distinct species within the T7-like lineage. Genome alignments showed extensive synteny across replication and structural modules, with major variability localized to the tail spike region, supporting the hypothesis that host range diversification in T7-like phages is primarily driven by recombination or modular exchange of receptor-binding proteins. Phylogenetic trees constructed from conserved marker genes (DNA polymerase, RNA polymerase, major capsid protein, tail spike protein, and terminase large subunit) consistently placed B1 within the *Autographiviridae* T7-like clade, closely related to Dev2, SH4, and IMM-002. Interestingly, B1 also showed phylogenetic proximity to *Shigella* phage SFP20, implying a shared ancestry followed by host-switching events, wherein divergence of tail fiber or tail spike genes enabled infection of *Enterobacter* instead of *Shigella*. Such modular adaptation is a recognized evolutionary mechanism among T7-like phages, facilitating ecological expansion while maintaining replication core conservation [41].

Analysis of GC skew [42] provided further support for B1’s classification as a T7-like phage. The alternating pattern of positive and negative GC skew across the genome indicates strand-specific nucleotide bias associated with transcriptional polarity, a feature characteristic of unidirectional replication in compact T7-like genomes. The absence of a strong polarity shift suggests that replication proceeds in a single direction from a terminal repeat region, consistent with DTR-type packaging strategies. Although no direct terminal repeats were detected, phylogenetic analysis of the terminase large subunit (ORF51) grouped B1 with DTR-type packaging phages, implying that it employs a similar mechanism for genome encapsidation. The apparent absence of short DTRs (typically 100–200 bp) could reflect assembly limitations, as these small terminal regions are often lost during sequencing or assembly collapse. Nevertheless, the conservation of DTR-type terminase architecture reinforces the functional and evolutionary continuity between B1 and classical T7-like phages.

Collectively, our results indicate that B1 represents a novel and evolutionarily distinct member of the T7-like *Autographiviridae*. Its genome encodes a unique combination of canonical and divergent features, including a SAM-cycle-associated metabolic gene (ORF1), a capsule-degrading tail spike (ORF47), and a dispersed lysis module (ORF21/48/50). These characteristics likely contribute to its ability to infect encapsulated MDR *E. cloacae* while sustaining efficient replication. The absence of tRNA genes, antibiotic resistance determinants, and known virulence factors further supports its genomic safety profile.

Although B1 displays properties desirable for therapeutic development—rapid replication, environmental stability, and a genome free of virulence or resistance genes—the present study did not evaluate in vivo efficacy, pharmacokinetics, or host immune interactions. Therefore, B1 should be regarded as a promising candidate for future preclinical studies rather than a validated therapeutic agent.

From a translational standpoint, the rapid adsorption, robust lytic activity, broad stability, and capsule-degrading capability of B1 suggest potential utility in phage-based strategies against MDR *E. cloacae*, including its incorporation into phage cocktails. In addition, the enzymatic activity of its tail spike may provide a basis for developing phage-derived depolymerases to assist antibiotic penetration or disrupt biofilms. Beyond translational relevance, the genetic distinctiveness of B1 expands current knowledge of *Autographiviridae* diversity and offers insight into how T7-like phages evolve through modular gene exchange and acquisition of metabolic innovations.

In summary, phage B1 represents a newly characterized T7-like lytic phage with distinctive genomic and functional adaptations that enhance its ability to infect encapsulated *Enterobacter* hosts. Its combination of evolutionary novelty, lytic efficiency, and favorable genomic features underscores its scientific value and highlights its potential in guiding future phage-based approaches targeting antibiotic-resistant pathogens.

## 4. Materials and Methods

### 4.1. Bacterial Strains and Cultural Condictions

A total of 128 clinical isolates of *E*. *cloacae* were obtained from the Department of Medical Research, Hualien Tzu Chi Hospital, Buddhist Tzu Chi Medical Foundation, Hualien, Taiwan. Additional bacterial strains were used for phage host range determination, including 10 *E*. *coli*, 8 *P*. *aeruginosa*, 5 *K*. *pneumoniae*, and 10 *A*. *baumannii* isolates. All bacterial strains were cultivated in Luria–Bertani (LB; Bio Basic Inc., Toronto, Canada) broth or on LB agar (LA; Bio Basic Inc.) plates at 37 °C under aerobic conditions. Bacterial lawns were prepared using the overlay agar technique with 0.7% agar [43].

### 4.2. Phage Isolation, Purification and Host Range Determination

The phage used in this study was isolated from sewage samples collected near Tzu Chi Hospital in Hualien, Taiwan. The β-lactam-resistant clinical isolate *E. cloacae* ECL51159 served as the host strain for phage isolation. Sewage samples were centrifuged at 8000× *g* for 10 min at room temperature, and the supernatant was subsequently filtered through a 0.45 μm syringe filter to remove debris and bacterial cells. The presence of lytic phages was verified by observing zones of clearing on bacterial lawns using spot assays or the double-layer agar method.

For phage purification, isopycnic centrifugation in a cesium chloride (CsCl) gradient was performed. High-titer phage lysate (10^12^ PFU) was first precipitated using 10% (*w*/*v*) PEG 8000 and 0.5 M NaCl at 4 °C for 12 h, followed by centrifugation at 15,000× *g* for 2 h at 4 °C using an Avanti JXN-26 centrifuge equipped with a JA-25.50 rotor (Beckman Coulter, Brea, CA, USA) to pellet the phage particles. The phage particles were resuspended in SM buffer (0.05 M Tris-HCl, pH 7.5, containing 0.1 M NaCl, 0.008 M MgSO_4_·7H_2_O, and 0.01% gelatin). The suspension was layered onto a CsCl step gradient with densities of 1.7, 1.5, 1.45, and 1.3 g/mL and centrifuged at 25,000 rpm for 3 h at 4 °C using an SW 41 Ti rotor in an Optima XPN-100 ultracentrifuge (Beckman Coulter). The visible phage band was carefully collected, dialyzed against SM buffer at 4 °C for 24 h, and stored at 4 °C until further use [43].

To assess the host range of the phage, a modified spot assay was carried out following previously described procedures [44]. Briefly, 200 μL of mid-log-phase bacterial culture was mixed with 5 mL of molten LB soft agar (0.7% agar) and poured onto LB agar (LA) plates. After solidification and drying for approximately 5 min at room temperature, 5 μL of the phage suspension (approximately 10^8^ PFU) was spotted onto the surface. The plates were incubated at 37 °C for 16 h, after which the formation of clear zones was observed. The presence of a clear spot indicated phage-induced lysis and confirmed that the bacterial strain was susceptible to the phage.

### 4.3. Morphological Observation Using Transmission Electron Microscopy (TEM)

Phage morphology was examined by TEM using negatively stained preparations. A 10 μL aliquot of the dialyzed phage suspension (approximately 10^10^ PFU) was applied onto a formvar-coated 300-mesh copper grid and stained with 2% uranyl acetate. The morphological features of the phage particles were then observed using a Hitachi H-7500 TEM (Hitachi High-Tech Corp., Tokyo, Japan) operated at an accelerating voltage of 80 kV and equipped with a CCD camera.

### 4.4. One-Step Growth and Adsorption Efficiency of Phage

The one-step growth curve of the phage was determined using a modified version of a previously described method [43]. A 5 mL culture of the host bacterium was grown to an OD_600_ of 0.6–0.8. Subsequently, 1 mL of the culture (approximately 1.0 × 10^8^ CFU) was harvested by centrifugation at 8000× *g* for 5 min at room temperature, resuspended in 0.9 mL of SM buffer, and mixed with 0.1 mL of phage suspension (1.0 × 10^5^ PFU/mL) to obtain an inoculum with an MOI of 0.001. The mixture was incubated on ice for 30 min to maximize adsorption and synchronize infection. Following incubation, cells were centrifuged at 8000× *g* for 2 min to remove unbound phages, and the pellet was resuspended in 15 mL of pre-warmed LB broth. The culture was incubated at 37 °C with shaking at 200 rpm, and samples were taken at 5-min intervals for up to 35 min. Phage titers were determined using the double-layer agar method. The burst size was estimated by dividing the increase in phage titer during the first lysis event by the estimated number of infected cells after the adsorption step. Because this modified protocol used a lower MOI than classical Delbrück conditions, the burst size represents an approximation based on the dominant initial replication cycle [45].

Phage adsorption efficiency was evaluated as described previously [43]. In brief, host bacterial cells were infected with the phage at a multiplicity of infection (MOI) of 0.001 and incubated at 37 °C with shaking. Take 100 μL of sample every minute for 10 min, centrifuge at 12,000 rpm for 5 min, and then analyze the supernatant using the double-layer agar method to quantify unadsorbed phages. The adsorption efficiency (%) was calculated using the following formula:

Adsorption efficiency (%) = (Initial phage titer − Unadorbed phage titer)/initial phage titer × 100

### 4.5. Bacteriolytic Curve Ominf the Phage

The in vitro bacteriolytic activity of the phage was evaluated using a microtiter plate liquid assay with slight modifications to a previously described method [46]. Briefly, the host bacterial suspension (~10^8^ CFU/mL) was prepared by diluting an overnight culture 1:100 in fresh LB broth and incubating until the OD_600_ reached approximately 0.3. Phage lysates were serially diluted in SM buffer resulting in multiplicities of infection (MOIs) of 0.01, 0.03, 0.1, and 1, respectively. For each assay, 180 μL of the bacterial culture was combined with 20 μL of phage suspension at varying concentrations in sterile, flat-bottom 96-well transparent microplates (Falcon^®^, Corning Inc., Corning, NY, USA). The plates were incubated at 37 °C with orbital shaking, and bacterial growth was monitored by measuring OD_600_ at 15 min intervals for 90 min using a Clariostar Plus microplate reader (BMG Labtech, Offenburg, Germany). The bacterial growth curves were generated by plotting OD_600_ values (after baseline correction) against time. All experiments were conducted in triplicate to ensure reproducibility.

### 4.6. The Influence of the Enviromental Factors on Phage Stability

Phage infectivity tests were performed as described by Jurczak-Kurek et al. with a minor modification [47]. To determine the infection activity of phage lysate, the following external factors were tested: temperature (4, 25, 37, 50, and 65 °C) and pH (3, 5, 7, 9, and 11). The phage lysate was diluted with LB (at the volume proportion 1:9) and incubated under the conditions described. The mixture was then withdrawn shortly, and serial 10-fold dilutions were used for double-layer agar plating. Phages without any treatment were the control. After overnight incubation at 37 °C, the titration of remaining plaque-forming phages was calculated.

### 4.7. Phage DNA Extraction, Genome Size Estimation, and Restriction Analysis

To extract phage DNA, the phage lysate was initially treated with DNase I (1 U/μL; Thermo Fisher Scientific Inc., Waltham, MA, USA) and RNase A (5 μg/μL; Thermo Fisher Scientific) at 37 °C for 30 min to degrade bacterial nucleic acids. Subsequently, a mixture containing 10 μg/mL proteinase K, 0.5 M EDTA, and 10% SDS was added for 3 h at 55 °C and then inactive by 70 °C to disrupt the viral capsid and inactivate DNase I and RNase A. The sample was then subjected to phenol–chloroform extraction twice. The phage DNA pellet was precipitated via centrifugation at 12,000 rpm for 30 min using 95% alcohol, and washed with 75% ethanol. The pellet was then resuspended in 30 μL of Tris-EDTA (TE) buffer [48]. The concentration of the extracted DNA was evaluated using nanodrop measurements performed on a NanoDrop^TM^ 2000C Spectrophotometer (Thermo Fisher Scientific).

To estimate the genome size of the phage, DNA was extracted and analyzed using a CHEF-DR III pulsed-field gel electrophoresis (PFGE) system (Bio-Rad Laboratories, Hercules, CA, USA). Electrophoresis was performed at 6 V/cm with pulse times ranging from 5 to 20 s for 10 h at 14 °C in 0.5× Tris-borate-EDTA (TBE) buffer. The genome size was determined by comparison with the MidRange PFG Marker (New England Biolabs, Ipswich, MA, USA).

For restriction pattern analysis, purified phage DNA was digested with specific restriction enzymes under conditions recommended by the manufacturer (Fermentas, Waltham, MA, USA). The resulting fragments were separated on a 0.8% agarose gel, and fragment sizes were estimated by comparison with the TriDye^TM^ 1 kb DNA Ladder and λ DNA–HindIII Digest markers (New England BioLabs Inc., Ipswich, MA, USA).

### 4.8. DNA Sequencing and Genome Analysis of Phage

The isolated phage genomic DNA (~5 μg) was sent to Allbio Life Co., Ltd. (Taichung, Taiwan) for genome sequencing, quality assessment of sequence reads, and de novo assembly. The phage DNA was fragmented down to a length of approximately 500 bp using the Covaris ultrasonic crusher (Covaris Inc., Woburn, MA, USA), and end repair was then performed before sticky end generation by adding base A to the 3′ end. Electroporation was employed for target fragment recovery, following PCR amplification of the DNA fragments flanked by adapters. The PCR products were cleaned and validated using Bioanalyzer (Agilent Technologies, Inc., Santa Clara, CA, USA). The qualified libraries were PE150 pair-end sequenced on the HiseqXten/Novaseq/MGI2000 System (Illumina, San Diego, CA, USA). The open reading frame was annotated using the RAST annotation server web [49] (ATG, GTG, CTG, and TTG were considered possible start codons; a >30 aa putative product length was required). Automatic annotation was manually reviewed using the BlastP algorithm against RefSeq proteins deposited in the GenBank database [50]. tRNA gene predictions were performed using ARAGORN [51] and tRNAscan [52]. Virulence factors and drug resistance of phage genome were compared using VirulenceFinder 2.0 and ResFinder 1.4 [53]. For relationships to other phages, the complete genome sequence of Aeromonas phage JELG-KS1 was first subjected to a BlastN search against the non-redundant NCBI Nucleotide database. Afterward, 42 top-scoring hits to genomes of other phages were downloaded and subjected to their pairwise intergenomic similarity calculations using VIRIDIC [26] under default settings. The whole-genome phylogenetic tree was constructed via the tree building online resource (VICTOR) [54]. The phylogenetic trees were generated in MEGA 12 using the neighbor-joining approach and 1000 bootstrap replications [55], and the proteome tree was generated based on the whole-genome sequence using VipTree. Moreover, GC skew analysis was performed on Proksee [56]. The B1 genome sequence with annotations was deposited in the GenBank database under the Submission ID: 3020046.

### 4.9. Analysis of Phage Structural Proteins by Liquid Chromatography–Tandem Mass Spectrometry (LC–MS/MS)

To analyze the structural proteins of the phage, CsCl-purified virion particles were mixed with lysis buffer containing 62.5 mM Tris-HCl (pH 6.8), 5% 2-mercaptoethanol, 2% sodium dodecyl sulfate (SDS), 10% glycerol, and 0.01% bromophenol blue. The mixture was boiled for 10 min and subsequently resolved by 12% SDS–polyacrylamide gel electrophoresis (SDS-PAGE). Protein bands were visualized using Coomassie Brilliant Blue staining. Selected protein bands were excised from the gel for in-gel digestion. The gel pieces were subjected to reduction and alkylation steps, followed by enzymatic digestion with trypsin (Promega, Madison, WI, USA). The resulting peptides were extracted from the gel fragments and analyzed using an UltiMate 3000 RSLCnano system coupled with a Q Exactive mass spectrometer (Thermo Fisher Scientific). The acquired MS data were searched against a custom database containing all theoretical peptide sequences predicted from the B1 phage genome to identify the virion-associated proteins.

### 4.10. Statistical Analysis

Statistical analysis of significance was conducted using GraphPad Prism 9 software. One-way analysis of variance and Dunnett’s multiple comparison tests were performed to assess the overall significance and compare specific groups, respectively. A significance level of *p* ≤ 0.05 was considered statistically significant. All experiments were repeated in triplicate to ensure reliability and consistency of the results.

## Figures and Tables

**Table 1:** Assignment of B1 genes.

ORF	Start	Stop	Length (aa)	Function	Functional Module	Hit Phage	Accession	Query Cover (%)	Identity (%)
1	948	1400	145	S-adenosyl-L-methionine lyase	Replication and transcription	Enterobacter phage IME278	QUE30078	100	96.0
2	1400	1513	37	hypothetical protein					
3	1491	1691	66	hypothetical protein					
4	1696	1821	41	hypothetical protein					
5	1893	2624	243	Protein kinase	Replication and transcription	Enterobacter phage EP1	UNA02567	100	84.8
6	2692	2853	53	hypothetical protein					
7	2856	3050	64	hypothetical protein					
8	3050	3376	108	hypothetical protein					
9	3480	6161	893	DNA-directed RNA polymerase	Replication and transcription	Enterobacter phage EP1	UNA02570	100	98.7
10	6180	6380	66	hypothetical protein					
11	6459	6929	156	hypothetical protein					
12	7037	7216	59	hypothetical protein					
13	7220	7480	86	hypothetical protein					
14	7485	8516	343	DNA ligase	Replication and transcription	Enterobacter phage vB_EholP_HHH	XFC52561	100	96.2
15	8632	8892	86	hypothetical protein					
16	8885	9313	142	Nucleotide kinase	Replication and transcription	Klebsiella phage Pkp-1	XRQ67095	100	95.8
17	9392	9550	52	Bacterial RNA polymerase inhibitor	Replication and transcription	Escherichia phage vB_EcoP_SP7	QLF80660	100	98.1
18	9607	10,305	232	Single-stranded DNA-binding protein	Replication and transcription	Enterobacter phage EP1	UNA02532	100	98.3
19	10,341	10,766	141	Endonuclease I	Replication and transcription	Enterobacter phage IME278	QUE30096	99	98.6
20	10,760	11,215	151	hypothetical protein					
21	11,205	11,663	152	endolysin	Lysis	Enterobacter phage vB_Ecl_MII_002	XGU09793	100	94.7
22	11,678	11,890	70	hypothetical protein					
23	11,959	13,665	568	DNA helicase/primase	Replication and transcription	Enterobacter phage vB_EholP_HHH	XFC52572	100	97.7
24	13,717	13,881	54	hypothetical protein					
25	13,953	16,124	723	DNA-directed DNA polymerase	Replication and transcription	Salmonella phage ST21	WJJ60283	100	97.7
26	16,134	16,448	104	hypothetical protein					
27	16,448	16,738	96	hypothetical protein					
28	16,735	16,944	69	hypothetical protein					
29	16,941	17,216	91	hypothetical protein					
30	17,209	18,075	288	exonuclease	Replication and transcription	Escherichia phage K1F	YP_338113	100	98.3
31	18,283	18,555	90	hypothetical protein					
32	18,565	18,789	74	hypothetical protein					
33	18,794	19,195	133	hypothetical protein					
34	19,188	19,439	83	hypothetical protein					
35	19,454	21,022	522	Portal protein	Structural and morphogenesis	Enterobacter phage EP1	UNA02548	100	99.4
36	21,127	22,008	293	Capsid assembly protein	Structural and morphogenesis	Enterobacter phage EP1	UNA02549	100	97.0
37	22,140	23,189	349	Major capsid protein	Structural and morphogenesis	Citrobacter phage SH4	YP_009279763	100	99.1
38	23,258	23,452	64	hypothetical protein					
39	23,510	24,076	188	Tail tubular protein A	Structural and morphogenesis	Citrobacter phage SH4	YP_009279764	100	98.4
40	24,076	24,303	75	hypothetical protein					
41	24,315	24,791	158	hypothetical protein					
42	24,757	27,126	789	Tail tubular protein	Structural and morphogenesis	Cronobacter phage GW1	YP_009820070	100	88.5
43	27,202	27,672	156	internal virion protein A	Structural and morphogenesis	Citrobacter phage SH4	YP_009279766	100	95.5
44	27,657	28,244	195	Internal virion protein B	Structural and morphogenesis	Enterobacter phage vB_EholP_HHH	XFC52595	100	95.4
45	28,256	30,538	760	Internal virion protein C	Structural and morphogenesis	Enterobacter phage IME278	QUE30119	100	98
46	30,544	34,440	1298	internal virion protein D	Structural and morphogenesis	Enterobacter phage IME278	QUE30120	100	97.8
47	34,508	36,670	720	Tail spike protein	Structural and morphogenesis	Cronobacter phage GW1	YP_009820026	31	63.4
48	36,711	36,905	64	Holin	Lysis	Cronobacter phage Dev2	YP_009005151	100	98.4
49	36,902	37,165	87	terminase small subunit	Structural and morphogenesis	Cronobacter phage Dev2	YP_009005152	100	100
50	37,268	37,717	149	i-spanin	Lysis	Shigella phage SFPH2	YP_009807458	100	98.7
51	37,714	39,477	587	Terminase, large subunit	Structural and morphogenesis	Cronobacter phage Dev2	YP_009005154	100	99.5
52	39,768	39,926	52	hypothetical protein					

## Data Availability

The original contributions presented in this study are included in the article. Further inquiries can be directed to the corresponding authors.
